# Macrophage regulated cell death: implications and mechanisms in organ transplantation

**DOI:** 10.3389/fimmu.2025.1604429

**Published:** 2025-05-23

**Authors:** Jincheng Hou, Chenghao Li, Geng Li, Wai Yen Yim, Bingchuan Geng, Yuqi Cheng, Zihao Wang, Zhengfeng Fan, Fuqiang Tong, Jiawei Shi, Yixuan Wang, Nianguo Dong

**Affiliations:** ^1^ Department of Cardiovascular Surgery, Union Hospital, Tongji Medical College, Huazhong University of Science and Technology, Wuhan, China; ^2^ Key Laboratory of Organ Transplantation, Ministry of Education, National Health Commission (NHC) Key Laboratory of Organ Transplantation, Key Laboratory of Organ Transplantation, Chinese Academy of Medical Sciences, Wuhan, China

**Keywords:** macrophage cell, regulated cell death (RCD), organ transplantation, ischemia-reperfusion injury, transplant rejection

## Abstract

Organ transplantation is a critical treatment for end-stage organ failure, but long-term graft survival remains suboptimal due to ischemia-reperfusion injury (IRI) and transplant rejection. The immune microenvironment, especially macrophages, plays a key role in these processes. Various forms of regulated cell death (apoptosis, autophagy, pyroptosis, ferroptosis, necroptosis) in macrophages significantly influence transplant rejection by mediating cellular communication and shaping the immune microenvironment. Apoptosis, pyroptosis, ferroptosis and necroptosis in macrophages exacerbate graft rejection while autophagy in macrophages protects against transplant rejection by reducing inflammation.This paper reviews the specific molecular mechanisms of macrophage regulated cell death, their impact on the IRI and transplant rejection, thus further provide potential therapeutic target for improving transplant outcomes.

## 1 Introduction

Organ transplantation provides a vital lifeline for thousands of patients with end-stage organ failure. Successful transplantation hinges on finding compatible donors and adhering to a regimen of multiple daily medications to prevent rejection. Advances in surgical techniques and immunosuppressive drugs have significantly reduced the incidence of short-term transplant rejection, resulting in over 90% of transplanted organs surviving the first year (1). Despite these advancements, long-term transplant survival rates remain unsatisfactory (2). Chronic transplant rejection poses a significant challenge as it often bypasses current immunosuppressive treatments. Additionally, ischemia-reperfusion injury (IRI), which occurs when blood supply returns to tissue after a period of ischemia, or lack of oxygen, triggers inflammation and damage. This condition is a major contributor to organ dysfunction and failure following resection and transplantation (3).

IRI is a two-stage pathological process occurring during organ transplantation. The initial ischemic injury is marked by ATP and glycogen depletion coupled with cellular metabolic stress induced by mitochondrial dysfunction, resulting in cell death. The subsequent reperfusion injury consists of severe damage occurring after blood flow and oxygen are restored (4). Oxidative stress, calcium overload, and excessive inflammatory responses are critical contributors to the pathogenesis of IRI. Introducing oxygen into the ischemic environment results in metabolic disturbances and generates large amounts of reactive oxygen species (ROS) and cytokines, or chemokines, which activate different immune cell subtypes to provoke a severe inflammatory response (5). Multiple studies suggest that IRI-induced tissue injury results in a series of pathological processes, including macrophage activation and inflammatory cytokine bursting, which contributes to graft dysfunction and even primary graft dysfunction.

Macrophages play a crucial role in transplant organ microenvironment. Tissue resident macrophages (TRMs) are also involved in IRI during transplantation. In thoracic transplants, TRMs in IRI trigger inflammation and recruit circulating immune cells, thus further amplifying the immune response. Research shows that macrophage proliferation, aggregation, and senescence-induced death impact rejection after transplantation (6). Macrophages can undergo regulated deaths like apoptosis, necroptosis, autophagy, pyroptosis, and ferroptosis, mediating cell signaling and shaping the immune microenvironment, which could influence transplant rejection (**Table 1**). Thus understanding macrophage and its related regulated death is essential for improving anti-rejection therapies in organ transplantation (7).

**Table 1:** Summary of the features, mechanisms, and impact of the different macrophage cell death pathways.

Types of Cell Death	Features	Mechanism	Functional outcomes
Macrophage apoptosis	1. biochemical features: Caspases activate oligonucleosomal DNA fragments. 2. morphological features: Membrane blebbing, reduction in cell and nuclear volume, nuclear fragmentation.	Signal pathways mediated by death receptors, mitochondria, endoplasmic reticulum, Caspases, P53, and Bcl-2	1. Increased ROS: NO, oxLDL, LPS 2. Increase inflammatory cytokines: TNF-α, IL-1β 3. Increased Cellular cytokines: IL-10, IL-6, PGE2
Macrophage autophagy	1. biochemical features: Increased lysosomal activity. 2. morphological features: Formation of double-membrane autophagolysosomes.	PI3K-AKT-mTOR, MAPK-ERK1/2-mTOR signaling pathways	1. Increased DAMP: HMBG1 2. Increased ROS: oxLDL, ATP 3. Increased Inflammatory cytokines: IL-6, IL-1β, IL-18 4. Increased Cellular cytokines: IL-10, TGF-β, TRAP
Macrophage pyroptosis	1. biochemical features: Dependent or independent of caspase-1 activation, GSDMD cleavage, and release of pro-inflammatory cytokines. 2. morphological features: Nuclear condensation, cell swelling, formation of pores in the cell membrane, cell collapse, and rupture.	The signaling pathway mediated by Caspase-1 and NLRP3	1. Increased DAMP: HMGB1 2. Increased ROS: ATP, K+ 3. Increased Inflammatory cytokines: NLRP3, ASC, TNF-α 4. Increased Cellular cytokines: IL-1α, IL-1β, IL-18, IL-8
Macrophage ferroptosis	1. biochemical features: Inhibition of xCT, reduction in GSH, inhibition of GPX4; accumulation of iron and lipid peroxidation. 2. morphological features: Mitochondrial shrinking, reduction or disappearance of mitochondrial cristae, and rupture of the mitochondrial outer membrane.	xCT and Gpx4, MVA, HSF1-HSPB1, p62-Keap1-Nrf2 pathways, LSH signaling pathway	1. Increased DAMP: HMGB1 2. Increased ROS: NO, iNOS, NO 3. Increased inflammatory cytokines: TNF-α, IL-1, IL-6 4. Increased Cellular cytokines: IFN-γ, TGF-β
Macrophage necroptosis	1. biochemical features: Activation of RIPK1, RIPK3, and MLKL. 2. morphological features: Cell swelling, membrane rupture, and release of cytoplasmic contents.	RIPK1/RIPK3; RIPK3	1. Increased inflammatory cytokines: TNF-α increase 2. Increased Cellular cytokines: IFN-γ

This review will focus on the various modes of regulated cell death of infiltrating macrophages and their specific role in IRI and transplant rejection. It will also provide potential therapeutic target for improving transplant outcomes.

## 2 Macrophages in transplantation

### 2.1 Macrophages play vital roles in transplant IRI

IRI is an inevitable pathological event during organ transplantation, occurring throughout the process from donor organ procurement and preservation to subsequent reperfusion in the recipient. IRI triggers a cascade of cellular and molecular changes, including mitochondrial dysfunction, excessive ROS generation, inflammatory cascade activation, and immune cell infiltration, ultimately leading to apoptosis, tissue damage, and impaired graft function (8).

An increasing body of evidence suggests that macrophages play a pivotal role in both the occurrence and regulation of IRI during organ transplantation. TRMs are the first to be stimulated by IRI and, upon activation, establish a highly pro-inflammatory microenvironment. These macrophages release damage-associated molecular patterns (DAMPs), further recruiting circulating immune cells and exacerbating local immune responses. For instance, studies on renal transplantation have demonstrated that massive macrophage infiltration following IRI leads to the secretion of inflammatory cytokines, promotion of neutrophil recruitment, and induction of apoptosis in graft tissues, thereby aggravating acute kidney injury (9). In liver transplantation, Kupffer cells (KCs), the tissue resident hepatic macrophages, recognize DAMPs and become activated early during IRI, subsequently releasing a substantial amount of pro-inflammatory cytokines, including IL-1, IL-6, IL-8, IL-12, and TNF-α, thereby driving sterile inflammation (10).

Macrophage activation induced by IRI is closely linked to the pattern recognition receptors (PRRs) activation on their surface. During the early post-transplant period, surgical trauma and IRI promote the release of DAMPs, which activate PRRs such as TLR4 and TLR9, further propagating the inflammatory cascade (7). In a murine liver transplantation model, the absence of TLR4 in donor organs significantly reduced IRI-associated injury, underscoring its critical role in the process (11). Additionally, Dectin-1, another PRR, is upregulated during IRI, and its activation promotes inflammatory macrophage polarization and induces the release of pro-inflammatory cytokines such as IL-1β and TNF-α (12).

### 2.2 Macrophages have critical roles in transplant rejection

Acute allograft rejection typically occurs within weeks to months post-transplantation and is primarily driven by the recipient’s immune system recognizing donor antigens, leading to the activation of alloreactive T cells (acute cellular rejection, ACR) or the production of donor-specific antibodies targeting donor HLA (antibody-mediated rejection, AMR). Studies have demonstrated that macrophages are the predominant infiltrating cells in allograft tissues during acute rejection (13). In biopsy samples from kidney transplant recipients, extensive monocyte infiltration is highly correlated with the severity of rejection (14). Furthermore, experimental models of ACR in mice have shown that inhibition of the M-CSF receptor reduces the proliferation of infiltrating macrophages, thereby mitigating the severity of rejection (15).

Macrophages mediate acute rejection through antigen presentation and interactions with adaptive immune system. In human heart transplant rejection, infiltrating monocytes express CD16, HLA-DR, and CD54, which are associated with T cell activation (16). Macrophage-derived IL-12 facilitates CD4+ T cell differentiation into Th1 or Th17 subsets, whereas regulatory macrophages secrete IDO to induce Treg differentiation (17). Th17 cells have been implicated in acute rejection and may serve as predictive biomarkers. In liver transplantation, CD16+ monocytes suppress Treg activity, promoting acute rejection, whereas TIM4 blockade in Kupffer cells (KCs) inhibits Th2 responses and enhances Treg generation, potentially facilitating immune tolerance (18, 19).

Chronic rejection (CR) represents a major barrier to long-term graft survival, typically occurring years post-transplantation. Its hallmark pathological features include chronic allograft vasculopathy (CAV) and interstitial fibrosis, ultimately leading to graft failure. Although T cell infiltration persists in CR, macrophages are the predominant infiltrating immune cells (20). In a murine CAV model of heart transplantation, macrophage depletion significantly prolongs graft survival and attenuates transplant vasculopathy independently of T and B cells (21). Additionally, clinical studies have demonstrated a strong correlation between macrophage presence and disease progression in chronic rejection biopsies from kidney transplant recipients (22).

The role of macrophages in chronic rejection extends beyond inflammation to tissue remodeling. In chronic renal allograft rejection, a subset of macrophages co-expresses CD68 (a macrophage marker) and α-SMA (a marker of smooth muscle cells and myofibroblasts), suggesting that macrophage-to-myofibroblast transition may contribute to graft fibrosis. Lineage-tracing studies have revealed that recipient monocyte-derived macrophages may account for up to half of the myofibroblasts within the graft, further confirming their critical role in transplant fibrosis (23).

## 3 Macrophage regulated cell death in transplantation

### 3.1 The role of macrophage apoptosis in transplantation

#### 3.1.1 Mechanisms of macrophage apoptosis

Apoptosis, or programmed cell death, is a tightly regulated process essential for normal development, homeostasis, and defense against external stressors within multicellular organisms (24). Unlike necrosis, apoptosis is marked by distinct morphological changes, including nuclear condensation and fragmentation of chromatin into 180–200 base pair fragments. During apoptosis, phosphatidylserine (PtdSer) flips from the inner to the outer leaflet of the cell membrane, signaling phagocytes to engulf the apoptotic bodies. Importantly, the cell membrane remains intact throughout this process, preventing inflammation in the surrounding tissue. This efficient clearance mechanism ensures that apoptosis proceeds without damaging neighboring cells or provoking an inflammatory response (25).

Macrophage apoptosis can be triggered by various factors, including oxidative stress, elevated concentrations of cytokines (such as TNF-α), activation of the Fas death pathway by Fas ligand (FasL), and endoplasmic reticulum (ER) stress (26). The extrinsic apoptotic pathway is activated through the binding of death ligands (such as FasL, TNF-α, and TRAIL) to their corresponding death receptors (such as Fas, TNFR1, and DR4/5). These receptors contain death domains (DD) on the cytoplasmic side of the cell membrane, which interact with other DD-containing proteins to form the apoptotic signaling complex. TNF-α initiates apoptotic signaling by binding to its receptor TNFR1, triggering the assembly of Complex I on the plasma membrane (27). This complex comprises TRADD, TRAF2, RIPK1, and cIAP1/2, and activates the NF-κB pathway to upregulate anti-apoptotic proteins such as cFLIP and cIAPs (28). However, under conditions of protein synthesis inhibition (e.g., via cycloheximide) or mitochondrial release of Smac/Diablo, cIAPs are either antagonized or degraded, leading to the disassembly of Complex I. The liberated RIPK1 then associates with FADD and caspase-8 in the cytoplasm to form Complex II (29). Activated caspase-8 subsequently cleaves and activates downstream effector caspases-3/7, which execute apoptosis by proteolyzing substrates including the DNA fragmentation factor (DFF/ICAD) and cytoskeletal regulators (e.g., gelsolin and ROCKI). This cascade drives DNA fragmentation, cell shrinkage, and apoptotic body formation, culminating in programmed cell death (30). When FasL binds to Fas or TRAIL binds to DR4/5, the Fas-associated death domain (FADD) is recruited, which in turn recruits and activates procaspase-8, converting it to active caspase-8, thereby initiating the downstream apoptotic cascade (31). Additionally, pro-apoptotic signals in macrophages can also be activated through the interaction of PAMPs with pattern recognition receptors (PRRs), as well as via ER stress pathways. These pathways are capable of activating caspase-9 and caspase-12, further amplifying apoptotic signaling. Persistent ER stress can induce the unfolded protein response (UPR), where CCAAT/enhancer-binding protein homologous protein (CHOP) has been identified as a critical pro-apoptotic effector in macrophages. CHOP promotes the release of calcium ions (Ca²^+^) from the ER, activating calcium/calmodulin-dependent protein kinase II (CaMKII). CaMKII facilitates mitochondrial uptake of calcium ions, altering mitochondrial membrane potential and subsequently triggering the release of mitochondrial pro-apoptotic proteins, such as cytochrome C. Cytochrome C, together with caspase-9 and apoptotic protease activating factor-1 (Apaf-1), forms the apoptosome, which further activates effector caspases, thereby accelerating the apoptotic process (32, 33).

The intrinsic pro-apoptotic pathway can be activated by ER stress, cellular stress, or DAMPs, which trigger the mitogen-activated protein kinase (MAPK) signaling pathway. Activation of the MAPK pathway can provide pro-apoptotic signals through c-Jun N-terminal kinase (JNK) or p38, or anti-apoptotic signals via extracellular signal-regulated kinase (ERK1/2), depending on the cellular microenvironment and stress conditions (34). Ultimately, both intrinsic and extrinsic apoptotic pathways lead to the activation of effector caspases (such as caspase-3, caspase-6, and caspase-7), inducing hallmark apoptotic features such as DNA fragmentation, membrane blebbing, and cell shrinkage, eventually culminating in macrophage apoptosis (35).

#### 3.1.2 Macrophage apoptosis contributes to IRI and transplant rejection

Macrophage apoptosis plays a crucial role in IRI during organ transplantation, primarily by exacerbating inflammatory responses and tissue damage. Substantial evidence suggests that during kidney transplantation, IRI induces macrophage recruitment, thereby enhancing the secretion of pro-inflammatory cytokines, which aggravate acute IRI injury. This process further facilitates neutrophil infiltration and promotes apoptotic cell death (36). Additionally, necrotic cell death triggered by IRI leads to the mitochondrial release of DAMPs, which activate inflammatory cascades and intensify tissue damage (37). Studies have demonstrated that the administration of macrophage apoptosis inhibitors (AIM) enhances the clearance of necrotic cells via kidney injury molecule-1 (KIM-1), effectively reducing delayed graft function (DGF) and improving graft survival in murine kidney transplantation models. Conversely, increased macrophage apoptosis has been closely associated with exacerbated DGF and early graft loss (38). Similarly, in liver transplantation, Kupffer cells (KCs) are significantly affected by IRI (39). Research indicates that gadolinium chloride (GdCl3) mitigates IRI-induced injury by inhibiting KC apoptosis, whereas KC activation leads to the secretion of pro-inflammatory cytokines such as TNF-α, further exacerbating graft dysfunction (40, 41). However, KCs also exhibit protective effects during IRI (42). For instance, upregulation of IL-10 has been shown to mitigate IRI in fatty liver transplantation (43). Additionally, Mesenchymal stem cells (MSCs) regulate the TLR4-ERK1/2-caspase-3 signaling pathway through prostaglandin E2 (PGE2), thereby inhibiting KC apoptosis and reducing IRI induced damage (44).

Macrophage apoptosis is a critical factor in transplant rejection, contributing to the amplification of inflammatory responses, graft damage, and inhibition of immune tolerance. Activated macrophages regulate transplant rejection by secreting various pro-inflammatory cytokines, including IL-1β, IL-6, and TNF-α. These cytokines promote immune cell recruitment, activate fibroblasts, and influence T-cell polarization, collectively intensifying the inflammatory response. Furthermore, the accumulation of inflammatory cytokines, increased oxidative lipid levels, and other microenvironmental changes within the graft regulate macrophage polarization and apoptosis (45). *In vitro* studies have demonstrated that TNF-α, nitric oxide (NO), hypoxia, elevated oxidized low-density lipoprotein (oxLDL) levels, and intracellular cholesterol accumulation can induce macrophage apoptosis (46). *In vivo* studies have further shown that macrophages internalizing modified lipoproteins undergo foam cell transformation, triggering endoplasmic reticulum stress and accelerating apoptosis (47, 48). These processes not only exacerbate transplant rejection but also promote neutrophil recruitment, amplify tissue damage, and suppress the anti-inflammatory functions mediated by M2 macrophages, ultimately leading to graft dysfunction.

Overall, macrophage apoptosis significantly influences both IRI and transplant rejection through multiple mechanisms, ultimately affecting graft survival and function. The upregulation of macrophage apoptosis amplifies tissue damage via pro-inflammatory signaling pathways, collaborates with necrotic cell death, and exacerbates graft dysfunction while simultaneously inhibiting the protective effects of M2 macrophages.

### 3.2 The role of macrophage autophagy in transplantation

#### 3.2.1 Macrophage autophagy and its mechanism

Autophagy, also known as type II regulated cell death, plays a crucial role in the onset and progression of numerous diseases. This process involves cells utilizing lysosomes or vacuoles to degrade damaged organelles and macromolecules, especially under conditions of nutrient deprivation, hypoxia, and exposure to reactive oxygen species. By doing so, cells obtain materials necessary for reconstruction, regeneration, and repair, thus making autophagy a compensatory and self-protective cellular catabolic pathway that helps maintain intracellular homeostasis (49).

Autophagy can be classified into three distinct forms based on its physiological functions and the pathways leading the lysosomal delivery: macroautophagy, microautophagy, and chaperone-mediated autophagy (50). Among these, macroautophagy, often simply referred to as autophagy, is the most common and extensively studied form. In macroautophagy, degradation materials are encapsulated by double-membrane structures, known as autophagosomes. The outer membrane of autophagosomes then fuses with the lysosomal or vacuolar membrane, releasing the enclosed material into the lysosome or vacuole. The material is subsequently hydrolyzed by a series of hydrolytic enzymes into small molecules such as amino acids, carbohydrates, fatty acids, nucleotides (51).

The autophagy mechanism in macrophages is regulated by multiple signaling pathways in a coordinated manner. Under nutrient deprivation, AMPK is activated, inhibiting mTORC1 activity, which subsequently releases the ULK complex, initiating the autophagy process. The ULK complex activates class III PI3K and phosphorylates Beclin-1, generating phosphatidylinositol-3-phosphate (PI3P), a critical step in phagophore membrane formation. The elongation of the phagophore membrane involves two ubiquitin-like conjugation systems. In the first system, ATG12 conjugates with ATG5, forming the ATG12-ATG5-ATG16L complex, which localizes to the phagophore membrane. In the second system, LC3 conjugates with phosphatidylethanolamine (PE) through ATG7, forming LC3-II, which further integrates into the phagophore membrane. During this process, misfolded proteins or damaged organelles are marked by ubiquitin chains, recognized by the p62 protein, and bind to LC3, ultimately forming the autophagosome. The outer membrane of the autophagosome fuses with the lysosome, forming the autolysosome, where its contents are degraded by lysosomal enzymes and recycled into nutrients and metabolic products for cellular reuse (52). Additionally, the autophagy process is influenced by multiple regulatory mechanisms. Inactivation of mTORC1 facilitates the translocation of transcription factor TFEB into the nucleus, initiating the expression of autophagy and lysosomal genes. Oxidative stress can activate the Nrf2 pathway, regulating the transcription of autophagy-related genes (53). Disruption of calcium homeostasis can also induce autophagy via ER stress, with CaMKKβ and DAPK playing key roles in this process (54).

#### 3.2.2 Macrophage autophagy alleviates IRI and transplant rejection

Macrophage autophagy plays a protective role in IRI by modulating inflammatory responses and macrophage polarization. IRI induces tissue damage and activates DAMPs, such as HMGB1, which binds to TLR2 on macrophages, thereby triggering the NADPH oxidase 2 (NOX2)-mediated autophagic pathway to generate ROS. This process leads to the deactivation of NF-κB p50/p65 while upregulating P50/P50 homodimers, thereby inhibiting the release of pro-inflammatory cytokines (55). Additionally, HMGB1 can selectively activate the TLR4 receptor, which subsequently activates the mechanistic target of rapamycin (mTOR), thereby suppressing NF-κB-mediated autophagy and hindering M2 polarization (54). Moreover, mitophagy, a form of selective autophagy, facilitates the clearance of damaged mitochondria, reducing the accumulation of mitochondrial-derived DAMPs (e.g., ROS and mitochondrial DNA), thereby preventing excessive inflammasome activation and limiting the release of pro-inflammatory cytokines such as IL-1β and IL-18 (56). Studies have demonstrated that macrophages deficient in ATG16L1 exhibit significantly elevated levels of pro-inflammatory cytokines, highlighting the critical role of autophagy in mitigating IRI-induced inflammation (57).

In the context of transplant rejection, macrophage autophagy plays a pivotal role in regulating antigen presentation and immune cell recruitment. Autophagy modulates the expression of MHC molecules in macrophages, promoting MHC-II upregulation while downregulating MHC-I expression, thereby reducing antigen presentation and attenuating T cell-mediated immune responses. Research has shown that in ATG5^-/-^ and ATG7^-/-^ macrophages, MHC-I expression levels are higher compared to autophagy-competent macrophages, suggesting that autophagy plays a crucial role in the lysosome-mediated degradation of MHC-I (58). Furthermore, oxidized low-density lipoprotein (oxLDL) can activate autophagy via the MAPK8/9 signaling pathway, thereby promoting MHC-II expression and enhancing anti-inflammatory immune responses (59). LC3-associated phagocytosis (LAP), a specialized autophagic pathway, facilitates the clearance of apoptotic cells through TIM4-mediated efferocytosis, thereby promoting the secretion of anti-inflammatory cytokines (IL-10 and TGF-β) while inhibiting the release of pro-inflammatory cytokines (IL-6 and IL-1β) (60). Additionally, the CD47/SIRPα signaling pathway inhibits macrophage phagocytosis, whereas DAMPs, CCL2, and IL-6 can induce M2 polarization via the NF-κB-dependent autophagic pathway, reducing the secretion of inflammatory mediators (61). Enhanced macrophage autophagy further promotes M2 polarization through the chemotactic secretion of tumor necrosis factor receptor-associated protein (TRAP), thereby mitigating transplant rejection.

In summary, macrophage autophagy exerts protective effects during organ transplantation through multiple mechanisms. Firstly, by eliminating damaged mitochondria, autophagy prevents the accumulation of mitochondrial DAMPs, thereby suppressing excessive inflammasome activation and reducing the release of pro-inflammatory cytokines. Secondly, autophagy promotes M2 polarization while inhibiting M1 polarization, thereby alleviating local inflammatory responses. Additionally, enhanced autophagic activity facilitates efferocytosis, efficiently clearing apoptotic cells, thereby promoting the secretion of anti-inflammatory cytokines and reducing inflammatory damage. Lastly, autophagy plays a crucial role in antigen presentation, as it downregulates MHC-I expression, thereby diminishing allograft immunogenicity and ultimately reducing transplant rejection.

### 3.3 The role of macrophage pyroptosis in transplantation

#### 3.3.1 Macrophage pyroptosis and its mechanism

Pyroptosis, also known as inflammatory necrosis, is a distinct form of regulated cell death dependent on the caspase family. This process is characterized by the maturation and release of inflammatory factors such as IL-1β and IL-18, which trigger inflammatory cascade reactions. The pyroptosis signaling pathway can be divided into two types: the classical caspase-1-dependent pathway and the non-classical caspase-4/5/11-dependent pathway. Pyroptosis is often induced by viral or bacterial infections as well as endogenous damage, playing a critical role in the host’s innate immune defense against intracellular pathogens. By responding to infections and endogenous danger signals, pyroptosis helps combat infections and maintain immune homeostasis (62).

In 2015, research teams led by Shao Feng and Vishva M Dixit independently discovered that caspase-1 and caspase-4/5/11 induce cell pyroptosis by cleaving a protein called Gasdermin-D (GSDMD). Upon cleavage by these caspases, GSDMD releases its N-terminal domain, which has membrane-binding activity and forms pores in the cell membrane. This pore formation leads to changes in osmotic pressure, cell swelling, and eventual membrane rupture (63). The pyroptosis pathway is primarily regulated by inflammasomes, which the NLRP3 inflammasome being the most studied. The NLRP3 inflammasome is a member of the NLR family, which plays a crucial role in enhancing the immune system’s ability to detect microbial infections, produce pro-inflammatory cytokines, and regulate immune and inflammatory responses. In the classical pathway of pyroptosis, specific stimuli activate NLRP3, which then recruits and activates caspase-1. Activated caspase-1 cleaves and activates inflammatory cytokines such as IL-1β and IL-18 and cleaves the N-terminal sequence of membrane-bound GSDMD, generating membrane pores and resulting in cell pyroptosis.

There are two distinct pathways for pyroptosis activation in macrophages. They regulate pyroptosis through two main mechanisms: the classical pathway and the non-classical pathway. In the classical pathway, macrophages activate the NLRP3 inflammasome, promoting the activation of Caspase-1, which subsequently cleaves GSDMD to generate GSDMD-NT. This fragment forms pores in the cell membrane, leading to cell swelling and death, while releasing inflammatory cytokines such as IL-1β and IL-18, which further amplify the inflammatory response (64). In the non-classical pathway, macrophages regulate pyroptosis via Caspase-8 and Granzyme B. Granzyme B activates Caspase-3, which cleaves GSDME, inducing pyroptosis and the release of inflammatory cytokines. Additionally, TAK1 in macrophages activates the MAPK pathway, which regulates pyroptosis by promoting inflammasome activation and IL-1 production (65). MicroRNA-155-3p and other molecules also play a role in macrophage pyroptosis by regulating GSDME expression, influencing the occurrence and regulation of pyroptosis (66).

#### 3.3.2 Macrophage pyroptosis contributes to IRI and transplant rejection

Macrophage pyroptosis plays a crucial role in IRI, primarily by inducing inflammatory responses and exacerbating organ transplant damage. During the acute phase of hepatic IRI, the expression of ApoA-1 is significantly downregulated, a change that specifically enhances pyroptosis in macrophages rather than hepatocytes. This amplification of pyroptosis is mediated through the TLR4-NF-κB signaling pathway, leading to the release of pro-inflammatory cytokines IL-1β and IL-18, thereby worsening tissue damage (67). Furthermore, isoflurane pretreatment has been shown to alleviate hepatic IRI, with its mechanisms involving the suppression of caspase-11 expression, a reduction in the production of typical pyroptosis markers, and inhibition of NF-κB nuclear translocation by lowering intracellular Ca2+ levels, thus preventing NLRP3 inflammasome activation (68). Similarly, in the context of ovarian transplantation, pyroptosis significantly affects the function of grafts during the processes of cryopreservation and transplantation (69). Research indicates that during IRI, the expression of ASC and NLRP3 inflammasomes, activation of caspase-1, and secretion of IL-1 and IL-18 are significantly upregulated, suggesting the central role of pyroptosis in the IRI-associated inflammatory response (70). Moreover, existing evidence points to the assembly of inflammasomes primarily occurring in fibroblasts and bone marrow-derived infiltrating cells rather than cardiomyocytes, further supporting the complex regulatory role of pyroptosis in transplant injury (71).

During transplant rejection, macrophage pyroptosis exacerbates immune rejection and promotes graft damage through various mechanisms. Pyroptosis induces the secretion of IL-1β and IL-18, further activating the NF-κB signaling pathway, promoting the release of endogenous ATP, and activating the NLRP3 inflammasome via the P2X7 receptor, thereby forming a persistent inflammatory response (72). Additionally, DAMPs and ROS released during pyroptosis further activate the immune system, intensifying the local inflammatory environment. ATP stimulates macrophages to secrete IL-8, thereby enhancing neutrophil recruitment and facilitating DC-mediated antigen presentation, leading to substantial T-cell infiltration (73). Simultaneously, ATP activates NK cells and CD4+ T cells, enhancing macrophage chemotaxis and inducing polarization toward the M1 phenotype, thereby amplifying pro-inflammatory responses (74). Notably, TNF-α triggers pyroptosis by binding to cell surface receptors, further promoting immune rejection. Moreover, IL-1α, IL-1β, ATP, and HMGB1 can all stimulate NLRP3 inflammasome activation, further exacerbating the pyroptosis process (75). These immune regulatory mechanisms interact, leading to the deterioration of graft damage and increasing the risk of acute rejection.

In conclusion, macrophage pyroptosis plays a significant role in both graft damage and rejection during organ transplantation. In IRI, it exacerbates transplant organ injury by inducing the secretion of pro-inflammatory factors and activating inflammasomes, while in rejection, it further exacerbates immune rejection by enhancing the local inflammatory microenvironment, promoting T-cell infiltration, and inducing immune cell chemotaxis.

### 3.4 The role of macrophage ferroptosis in transplantation

#### 3.4.1 Macrophage ferroptosis and its mechanism

Ferroptosis is a novel form of iron-dependent regulated cell death characterized by the lethal accumulation of reactive oxygen species (ROS) due to the impairment of the glutathione (GSH)-dependent lipid peroxidation repair system (76). This type of cell death is intricately linked to various human diseases, including cancer, cardiovascular diseases, and neurodegenerative disorders. The core mechanism of ferroptosis involves the depletion of glutathione, decreased activity of glutathione peroxidases (GPXs)—with the reduction of GPX4 activity being particularly critical—and the resulting impairment of the GPX4-dependent cellular antioxidant capacity. Lipid peroxides that cannot be metabolized by the GPX4-catalyzed glutathione reductase reaction undergo Fe^2+^-mediated lipid oxidation, generating ROS and leading to oxidative cell death, known as ferroptosis. Ferroptosis significantly differs from other forms of regulated cell death. Morphologically, it is characterized by increased mitochondrial membrane density and mitochondrial contraction, without reductions in nuclear volume or chromatin condensation (77). Biochemically, ferroptosis is marked by increased lipid peroxidation, elevated ROS levels, disruption of the oxidative-antioxidative balance, loss of cell integrity, and cell death. These processes can be inhibited by antioxidants and iron chelators (78).

The mechanism of ferroptosis in macrophages involves excessive iron accumulation and the oxidative stress it induces. Iron overload can generate large amounts of ROS through the Fenton reaction, leading to lipid peroxidation and membrane damage, thereby triggering ferroptosis (79). Sources of iron include endogenous iron (such as free iron released from heme degradation) and exogenous iron (such as ferric iron absorbed through the transferrin-transferrin receptor 1 pathway). Under normal conditions, iron homeostasis is maintained by several mechanisms. Hepcidin regulates iron excretion by degrading ferroportin (FPN), heme oxygenase-1 (HO-1) releases iron through the degradation of heme, and NCOA4 increases intracellular free iron by promoting ferritin (FT) degradation (80). Additionally, iron metabolism in macrophages is regulated by different subtypes: M1 macrophages typically exhibit high levels of iron storage (due to high expression of ferritin and low expression of ferroportin), whereas M2 macrophages show lower iron storage (due to high expression of ferroportin and low expression of ferritin) (81). Iron overload can also influence macrophage polarization, promoting the inflammatory characteristics of M1 macrophages and potentially facilitating ferroptosis (82).

#### 3.4.2 Macrophage ferroptosis contributes to IRI and transplant rejection

Macrophage ferroptosis, through the activation of the HMGB1/TLR4 signaling axis and oxidative stress cascades, significantly exacerbates IRI during organ transplantation. Ischemia-induced upregulation of HMGB1 synergizes with the TLR4 receptor to drive macrophage activation, leading to the release of excessive inflammatory cytokines (TNF-α, IL-1, IL-6) and ROS, which directly cause damage to the graft parenchymal cells (83, 84). In both cardiomyocytes and non-cardiomyocytes, iron overload establishes a positive feedback loop with the inhibition of the mTOR signaling pathway, which, through mTOR-mediated ROS generation mechanisms, intensifies the process of cell death (85). Experimental evidence shows that mitochondrial-targeted overexpression of GPX4 significantly mitigates IRI through a dual protective mechanism: on one hand, it reduces the release of ferroptosis markers such as creatine kinase, and on the other, it decreases mitochondrial lipid peroxidation and improves contractile function (86). Notably, the ferroptosis inhibitor Ferrostatin-1 exhibits a selective protective effect in a heart transplant model, reducing the levels of oxidized phosphatidylethanolamine, selectively decreasing cardiomyocyte and fibroblast death, while endothelial cells remain unaffected.

Macrophage ferroptosis deeply contributes to the pathological process of transplant rejection through immune regulatory network imbalance and the collapse of antioxidant defense systems. The release of ferroptosis-related DAMPs mediates the diversity regulation of TP53 and downregulation of NCOA4 expression (87).The M1 phenotype maintains resistance to ferroptosis through iNOS/NO antioxidant pathways (88), while also activating CD8+ T cells via cell contact, inducing IFN-γ secretion (89), and leading to JAK/STAT1-mediated inhibition of the Xc^−^ system and ACSL4-driven lipid peroxidation (90, 91). Notably, M1 macrophages enhance the ROS/Fenton reaction through a positive feedback loop, accelerating the ferroptosis process in the graft microenvironment (92). In contrast, M2 macrophages, through the secretion of TGF-β/IL-6, inhibit the transcription of SLC7A11 and the function of GPX4, while suppressing CTL activation and inadvertently exacerbating antioxidant system dysfunction (93). This polarization imbalance results in a pro-inflammatory and pro-oxidative graft microenvironment, ultimately triggering an immune rejection cascade.

In conclusion, macrophage ferroptosis has profound implications in organ transplantation, particularly in IRI and transplant rejection. Ferroptosis exacerbates inflammation and graft damage by releasing DAMPs, promoting macrophage polarization, and increasing ROS production. Iron overload and the disruption of antioxidant defenses are key mechanisms underlying its role in IRI, while in transplant rejection, the activation of M1 macrophages and the enhancement of CTLs serve as critical pathogenic pathways of ferroptosis. In contrast, M2 macrophages display some immunosuppressive effects, but may also exacerbate graft damage by impairing the antioxidant system.

### 3.5 The role of macrophage necroptosis in transplantation

#### 3.5.1 Macrophage necroptosis and its mechanism

Necroptosis, also referred to as programmed necrosis, is a crucial mode of regulated cell death first described by Degterev et al. in 2005 (94). This process exhibits characteristic necrotic morphological features, including increased cell volume, organelle swelling, and plasma membrane rupture. From a physiological and biochemical perspective, necroptosis generates substantial amounts of ROS and pro-inflammatory factors, often resulting in the formation of necroptotic bodies (95).

Unlike typical necrosis, necroptosis is a regulated form of cell death governed by a distinct signaling pathway. Receptor-interacting protein kinases (RIPs), particularly RIPK1 and RIPK3, are essential signaling molecules that regulate cell death or survival during necroptosis. The expression levels of these kinases can indicate the extent of necroptosis (96).

The classical necroptosis pathway is initiated by the binding of TNF-α to membrane receptors, which transmits signals to RIPK1 and RIPK3. These kinases phosphorylate each other, gaining kinase activity, and subsequently phosphorylate the downstream mixed lineage kinase domain-like protein (MLKL). Activated MLKL oligomerizes and forms selective ion channels, ultimately leading to cell membrane rupture and necroptotic cell death. Additionally, when apoptosis is inhibited, necroptosis can serve as an alternative pathway to mediate cell death.

Necroptosis in macrophages can be induced by various factors, including TNF-α, TNF-related apoptosis-inducing ligand (TRAIL), Fas ligand (FasL), interferons, Toll-like receptor ligands, and virus-activated signaling pathways (97). Currently, TNF-α-induced necroptosis is the most extensively studied necroptotic pathway. Upon binding of TNF-α to trimeric TNFR1, TNFR1-associated death domain protein (TRADD) and receptor-interacting protein kinase 1 (RIPK1) are recruited, forming complex I. Subsequently, TNFR-associated factor 2 (TRAF2), TRAF5, cellular inhibitor of apoptosis protein 1 (cIAP1), and cIAP2 are recruited to TRADD, further stabilizing the formation of complex I. When RIPK1 undergoes deubiquitination and caspase-8 activity is inhibited, RIPK1 dissociates from complex I, enters the cytoplasm, and binds to RIPK3, facilitating the phosphorylation of both. Phosphorylated RIPK3 subsequently recruits and phosphorylates MLKL. Phosphorylation of MLKL induces its oligomerization, and the oligomerized MLKL translocates to the cell membrane, where it forms pores, leading to dysregulation of sodium and calcium ion channels, thereby executing necroptosis (98, 99).

#### 3.5.2 Macrophage necroptosis contributes to IRI and transplant rejection

Macrophage necroptosis significantly exacerbates IRI-related tissue remodeling following organ transplantation through a lipid deposition-fibrosis cascade. Studies have shown that in a mouse adipose tissue transplantation model, increased fibrosis post-transplantation is accompanied by macrophage aggregation around apoptotic adipocytes or large lipid droplets, forming typical crown-like structures (CLS) within fibrotic deposits. Within these CLS, lipid accumulation in macrophages promotes the formation of multinucleated giant cells (MFCs), which trigger necroptosis (100). Necroptosis, through a paracrine mechanism, induces the release of inflammatory cytokines and chemokines, thereby altering the expression of type I and type VI collagen in fibroblasts. These changes may contribute to the exacerbation of fibrosis and post-transplant organ damage.

Macrophage necroptosis significantly enhances the intensity and persistence of transplant rejection through multidimensional immune regulatory mechanisms. Macrophages modulate immune responses through their necroptotic effector molecules, RIPK1 and RIPK3 (101). The necroptosis-induced release of chemokines and ATP attracts large numbers of macrophages, prompting them to release cytokines such as TNF. TNF, by binding to the cell surface TNFR1 receptor, initiates further necroptosis and establishes a positive feedback loop, amplifying the inflammatory response. At the transcriptomic level, TNF-induced necroptosis interacts with the pro-inflammatory NF-κB pathway, further increasing the expression of pro-inflammatory factors (102). Additionally, when APCs engulf cells undergoing necroptosis, it directly promotes the maturation of DCs, enhancing their antigen-presenting capacity to CD8+ T cells and stimulating the production of IFN-γ, thereby strengthening the immune response and promoting the occurrence of transplant rejection (103).

In summary, macrophage necroptosis exacerbates transplant rejection and graft damage through multiple mechanisms. Necroptosis leads to macrophage aggregation into crown-like structures (CLS) and the formation of multinucleated giant cells (MFCs), thus promoting fibrosis. Furthermore, macrophages regulate the immune microenvironment through RIPK1 and RIPK3, with the release of TNF activating TNFR1 and amplifying the pro-inflammatory response via the NF-κB signaling pathway. Additionally, macrophage necroptosis enhances dendritic cell maturation and their antigen presentation to CD8+ T cells, thereby amplifying the immune response and aggravating transplant rejection.

## 4 Drug interventions targeting macrophage regulated cell death in transplantation

Targeted therapies that influence apoptosis, autophagy, ferroptosis, pyroptosis, and necroptosis can profoundly impact disease progression by modulating regulated cell death pathways.

### 4.1 Recent advances in drugs targeting apoptosis

Targeted regulators of apoptosis exhibit multidimensional therapeutic potential in inflammatory diseases by modulating both extrinsic and intrinsic signaling pathways. TNF-α antagonists such as HUMIRA (Adalimumab) and Enbrel (Etanercept) neutralize TNF-α signaling to inhibit excessive macrophage apoptosis, an effect that has been validated in autoimmune disease models, demonstrating the alleviation of tissue inflammation and the maintenance of immune homeostasis (30). The pan-caspase inhibitor IDN-6556 irreversibly inhibits caspase activity and has shown dual anti-inflammatory and anti-fibrotic effects in chronic hepatitis and liver transplant IRI models. Currently, it is undergoing a phase II clinical trial for liver transplant IRI. The anti-apoptotic Bcl-2 family inhibitor ABT-263 targets Bcl-2, Bcl-XL, and Bcl-w to modulate the apoptotic threshold of lymphocytes, potentially aiding in the clearance of aberrant lymphocytes in autoimmune diseases (104). IAP antagonists such as SM-406 promote the degradation of cIAP1/2, thereby enhancing caspase-8-mediated extrinsic apoptosis. In autoimmune disease animal models, SM-406 has been shown to facilitate neutrophil apoptosis at inflammatory sites and accelerate the resolution of inflammation (105). Additionally, the TRAIL receptor agonist Dulanermin induces apoptotic activation through TRAIL receptors, demonstrating potential in suppressing pathological immune infiltration in animal models (106). While drugs targeting apoptotic pathways show promise in inflammatory diseases, their clinical application requires caution due to potential risks such as infection susceptibility or tumorigenesis caused by excessive apoptosis suppression. Future strategies must achieve a precision balance in immune homeostasis to optimize therapeutic efficacy while minimizing adverse effects.

### 4.2 Recent advances in drugs targeting autophagy

Macrophage autophagy plays a protective role in the progression of organ transplantation by suppressing inflammatory responses, mitigating oxidative stress, and promoting cholesterol efflux, thereby providing a novel therapeutic avenue post-transplantation. mTOR inhibitors, as classical autophagy inducers, are represented by Everolimus, which significantly inhibits inflammation in animal models through mTOR suppression. This effect may be associated with the inhibition of macrophage inflammatory phenotypic transformation (107). Sirtuin family proteins, particularly Sirt1, are critical regulatory factors involved in autophagy and exert protective effects in cardiovascular diseases. The Sirt1 activator resveratrol (RSV) has been demonstrated to enhance macrophage autophagy and suppress inflammatory responses in cardiovascular disease models (108). Novel autophagy activators such as adiponectin (ADIPOQ) and 2-aminoacrylate derivatives (W09) enhance autophagic activity via the STK11/LKB1-AMPK-ULK1 and EGFR-RAS-RAF1-MAPK pathways, respectively, offering new perspectives on the metabolic-immune crosstalk regulation (109). Furthermore, chloroquine (CQ) and its derivative hydroxychloroquine (HCQ), as clinically approved autophagy inhibitors, block the fusion of autophagosomes and lysosomes, thereby inhibiting the late stage of autophagy. In contrast, PI3K inhibitors such as 3-methyladenine (3-MA) target the initiation phase of autophagy (110, 111). The dual regulatory nature of autophagy necessitates highly precise intervention. Overactivation may trigger cellular energy exhaustion or autophagic cell death, whereas excessive inhibition can exacerbate inflammation or metabolic dysregulation. Current pharmacological agents are limited by pathway crosstalk and dose-dependent effects. Future advances demand organ-selective drug delivery systems and real-time biomarker monitoring to guide dynamic modulation.

### 4.3 Recent advances in drugs targeting pyroptosis

Inhibitors targeting GSDMD-mediated pyroptosis have garnered significant attention due to their potential in inflammatory disease treatment. Recent studies have identified human Cys191 or murine Cys192 as key targets for small-molecule inhibitors such as Disulfiram (112), Necrosulfonamide (113, 114), and Fumarate (115), which suppress GSDMD-N oligomerization and membrane pore formation via covalent modifications or hydrogen bonding, thereby inhibiting pyroptosis. Additionally, LDC7559 inhibits neutrophil extracellular trap (NET) formation by targeting GSDMD-N activity, thereby suppressing pyroptosis (116). A newly identified compound, GI-Y1, inhibits pyroptosis in cardiac diseases by targeting the GSDMD-Arg7 site to prevent GSDMD-N pore formation on the cell membrane (117). Notably, DMB functions as a selective GSDMD activator, promoting GSDMD pore formation and pyroptosis through direct modification of Cys191, independent of GSDMD cleavage (118). Although GSDMD inhibitors block pyroptosis by targeting its core mechanism, challenges persist, including off-target effects due to broad cysteine-targeting specificity and crosstalk with other cell death pathways. Current research predominantly focuses on acute inflammatory models, leaving the dynamic regulatory mechanisms of pyroptosis in chronic diseases and the long-term safety of its inhibition unvalidated. Future efforts should integrate structural biology-driven optimization to enhance target selectivity.

### 4.4 Recent advances in drugs targeting ferroptosis

Ferroptosis modulators hold promise in organ transplantation therapy by interfering with intracellular iron metabolism and redox homeostasis. Ferroptosis activators such as ammonium ferric citrate induce ferroptosis by increasing intracellular iron load (119). The nuclear receptor coactivator 4 (NCOA4) exacerbates iron accumulation by promoting ferritin degradation, a mechanism confirmed in macrophage foam cell models to drive lipid peroxidation (120). Small-molecule compounds such as BAY 11–7085 activate the Nrf2-SLC7A11-HO-1 signaling axis to upregulate heme oxygenase-1 (HO-1), thereby promoting free iron release and enhancing ferroptosis sensitivity (121). Additionally, Siramesine and Lapatinib induce ferroptosis by disrupting iron uptake/export homeostasis through the regulation of transferrin receptors and membrane iron transporters (122). In contrast, ferroptosis inhibitors such as Ferrostatin-1 mitigate ferroptosis by scavenging reactive oxygen species (ROS) and reducing intracellular iron levels (123), while Curcumin exerts ferroptosis suppression via iron chelation, limiting iron accumulation (124). Drugs inhibiting ferroptosis protect grafts from ferroptotic damage but may impair immune cell clearance functions, increasing infection or tumor risks. Additionally, long-term intervention could disrupt iron metabolic homeostasis, potentially leading to conditions such as anemia. Future therapeutic designs must balance pathological cytoprotection with metabolic equilibrium to ensure clinical benefits outweigh risks.

### 4.5 Recent advances in drugs targeting necroptosis

Targeted inhibitors of necroptosis have demonstrated therapeutic potential in inflammatory diseases and ischemic injury by modulating receptor-interacting protein kinase (RIPK) signaling pathways. RIPK1 inhibitors, such as Necrostatin-1 (Nec-1) and its high-specificity derivative Nec-1s, block necroptotic signaling by stabilizing the inactive conformation of RIPK1’s kinase domain. This mechanism has been shown to attenuate tissue damage in models of atherosclerosis and myocardial IRI (94, 125, 126). The clinical-stage compound GSK2982772, a selective RIPK1 inhibitor, suppresses TNF-α-mediated inflammatory cytokine production and is currently undergoing phase IIa clinical trials for psoriasis and rheumatoid arthritis (127). Notably, the B-Raf inhibitor Dabrafenib exhibits off-target inhibition of RIPK3 through ATP-competitive binding, providing a novel strategy for necroptosis modulation (128). Initially identified as a cysteine-reactive molecule targeting MLKL to inhibit necroptosis, Necrosulfonamide was later found to suppress pyroptosis as well (129). Necroptosis inhibitors selectively block RIPK3-MLKL signaling but risk interfering with RIPK1’s physiological roles in apoptosis and inflammatory signaling, potentially compromising immune surveillance or increasing infection susceptibility. Multitarget inhibitors may further induce metabolic or proliferative abnormalities through off-target effects. Future development should prioritize structure-guided optimization and synergistic pathway regulation to preserve tissue repair capacity while avoiding unintended consequences.

Targeting macrophage cell death pathways offers a novel therapeutic direction in the field of organ transplantation (**Table 2**). By modulating distinct cell death mechanisms—including apoptosis, autophagy, pyroptosis, ferroptosis, and necroptosis—these strategies enable precise intervention in key pathological processes such as transplant-associated inflammation, IRI, and immune rejection. Beyond acting on specific cell death pathways, these approaches can also synergistically regulate the metabolism-immune network, thereby reshaping microenvironmental homeostasis. This paradigm holds promise for extending graft survival and mitigating rejection, potentially establishing a new framework for transplantation immunotherapy.

**Table 2:** Summary of drugs and targets based on regulated cell death.

Types of Cell Death	Drug Name	Effects on cell death	Target Sites of Action	Clinical Trial Progress	References
Macrophage apoptosis	HUMIRA (Adalimumab)	Antagonist	TNF-α	Approved	(30)
	Enbrel (Etanercept)	Antagonist	TNF-α	Approved	(30)
	IDN-6556	Antagonist	Pan-caspase	Phase II clinical trial	(104)
	ABT-263	Agonist	Bcl-2, Bcl-XL, Bcl-w	Phase I clinical trial	(104)
	SM-406	Agonist	cIAP1/2	Phase I clinical trial	(105)
	Dulanermin	Agonist	TRAIL	Phase II clinical trial	(106)
Macrophage autophagy	CQ/HCQ	Antagonist	Autophagosomes and lysosomes	Approved	(110)
	3-MA	Antagonist	PI3K	Preclinical study	(111)
	Everolimus	Agonist	mTOR	Preclinical study	(107)
	Resveratrol	Agonist	Sirt1	Preclinical study	(108)
	ADIPOQ	Agonist	STK11/LKB1-AMPK-ULK1	Preclinical study	(109)
	W09	Agonist	EGFR-RAS-RAF1-MAPK	Preclinical study	(109)
Macrophage pyroptosis	Disulfiram	Antagonist	human GSDMD-Cys191 or mouse GSDMD-Cys192 site	Preclinical study	(112)
	Necrosulfonamide	Antagonist	human GSDMD-Cys191 or mouse GSDMD-Cys192 site	Preclinical study	(113, 114)
	Fumarate	Antagonist	human GSDMD-Cys191 or mouse GSDMD-Cys192 site	Preclinical study	(115)
	LDC7559	Antagonist	GSDMD-N	Preclinical study	(116)
	GI-Y1	Antagonist	GSDMD-Arg7	Preclinical study	(117)
	DMB	Inducer	Cys191	Preclinical study	(118)
Macrophage ferroptosis	Ferrostatin-1	Antagonist	ROS	Preclinical study	(123)
	Curcumin	Antagonist	Iron chelation	Preclinical study	(124)
	Ammonium ferric citrate	Agonist	Increase iron load	Preclinical study	(119)
	NCOA4	Agonist	Increase iron load	Preclinical study	(120)
	BAY 11-7085	Agonist	Nrf2-SLC7A11-HO-1	Preclinical study	(121)
	Siramesine and Lapatinib	Agonist	Transferrin receptor and membrane iron transporter	Preclinical study	(122)
Macrophage necroptosis	Necrostatin-1	Antagonist	RIPK1	Preclinical study	(94, 125, 126)
	GSK2982772	Antagonist	RIPK1	Phase II clinical trial	(127)
	Dabrafenib	Antagonist	RIPK3	Approved	(128)
	Necrosulfonamide	Antagonist	MLKL	Approved	(129)

## 5 Conclusion

Macrophage regulated cell death is fundamentally associated with the occurrence and progression of transplant rejection, playing a critical role in its development and revealing it as a potential therapeutic target for vascular diseases. Despite the growing recognition of this association, many specific mechanisms remain unclear. The microenvironment of transplant rejection is particularly complex, with multiple macrophage death-inducing factors coexisting and resulting in diverse modes of macrophage death. The intricate interrelationships among these different types of cell death necessitate a comprehensive approach. Furthermore, each mode of macrophage death exerts distinct effects on transplant rejection, requiring tailored strategies for different stages and patterns of rejection.

**Figure 1:**
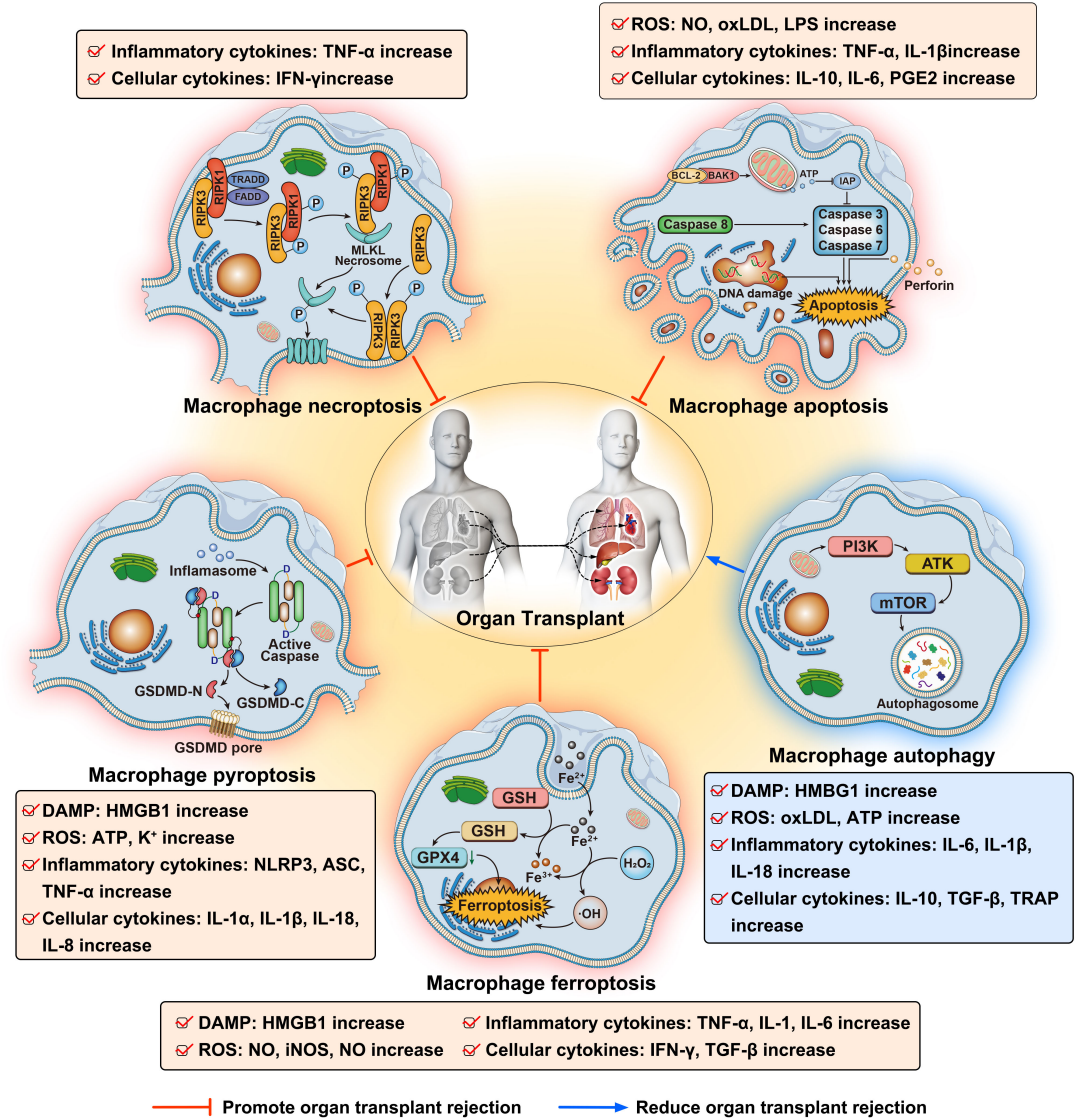
Impact of macrophage regulated cell death in organ transplantation.

In the various processes of RCD in macrophages, there are interconnections and mutual regulations among different modes of cell death. TNF-α-mediated apoptosis and necroptosis share upstream signaling nodes: both pathways are initiated by TNFR1 activation and the formation of Complex I, with RIPK1 acting as a hub molecule in the assembly of Complex II. However, the divergence between these pathways hinges on caspase-8 activity and the proteolytic status of RIPK1. In apoptosis, sufficient caspase-8 activity cleaves and inactivates RIPK1, driving caspase-3/7-dependent substrate degradation, ultimately leading to non-inflammatory cell clearance via apoptotic body formation. Conversely, in necroptosis, insufficient caspase-8 activity prevents RIPK1 cleavage, enabling RIPK1 to engage RIPK3 via its RHIM domain and initiate a RHIM-dependent RIPK3-MLKL phosphorylation cascade. This cascade culminates in MLKL oligomerization, plasma membrane rupture, and a pro-inflammatory necrotic death. This signaling bifurcation mechanism underscores cellular flexibility in dynamically modulating caspase activity to select death modalities under stress: apoptosis maintains tissue homeostasis, while necroptosis serves as an inflammatory backup mechanism to counter infections or pathological insults. Among the various forms of regulated cell death in macrophages, apoptosis, pyroptosis, ferroptosis, and necroptosis significantly promote organ transplant rejection, leading to graft dysfunction and adversely affecting the survival time of transplant patients. These cell death pathways induce strong inflammatory responses and activate immune cells, disrupting the normal function of the graft and ultimately triggering the occurrence and development of rejection. In contrast, the process of autophagy in macrophages exhibits an opposite effect. Autophagy in macrophages mitigates transplant rejection and graft damage by clearing damaged mitochondria and reducing the accumulation of damage-associated molecular patterns (DAMPs), thereby suppressing excessive inflammasome activation and cytokine release. Additionally, autophagy promotes the anti-inflammatory M2 phenotype, enhances the clearance of apoptotic cells, and modulates antigen presentation by reducing MHC-I expression, all of which contribute to decreased local inflammation and protection of the graft (**Figure 1**).Therefore, in-depth research on the impact of different forms of regulated cell death in macrophages on the immune response to organ transplantation, and reducing macrophage death pathways that cause graft damage, are of paramount importance for improving the success rate of organ transplants and extending patient survival time.

The limitation of this article is that regulatory cell death (RCD) includes various forms such as apoptosis, autophagy, ferroptosis, pyroptosis, necroptosis, cuproptosis, parthanatos, NETosis, and entosis. However, due to the limited research on cuproptosis, parthanatos, NETosis, and entosis in organ transplantation, these forms were not included in this article. We hope that future studies will further investigate the roles of these other forms of regulatory cell death in organ transplantation.

Interventional studies targeting RCD pathways in macrophages remain exploratory in the field of transplantation. Although emerging evidence suggests that modulating macrophage apoptosis, pyroptosis, autophagy, ferroptosis, or necroptosis may influence transplant immunity, animal studies directly targeting macrophage RCD pathways via genetic editing or pharmacological interventions—with systematic evaluation of transplant outcomes such as graft survival and immune cell infiltration dynamics—are still limited. The future development of mechanistic animal models will accelerate translational research, providing a robust theoretical foundation for devising macrophage RCD-targeted immunomodulatory strategies in transplantation.

While this review focuses on the role of recipient-derived infiltrating macrophages in transplant rejection, the impact of donor-derived TRMs on transplant outcomes warrants careful consideration. As innate immune components inherently residing within the graft, donor TRMs participate in transplant pathophysiology through the following mechanisms: IRI activates donor TRMs to release pro-inflammatory cytokines such as IL-1β and TNF-α, triggering neutrophil recruitment and exacerbating tissue damage. Donor TRMs migrate to recipient secondary lymphoid organs carrying graft-specific antigens, where they directly activate alloreactive T cells via MHC-II-mediated direct allorecognition, a process critical for initiating adaptive immune responses. In long-term surviving grafts, donor TRMs promote angiogenesis through growth factors like VEGF and PDGF, yet their overactivation may also drive TGF-β/Smad3-dependent fibrosis, contributing to chronic allograft dysfunction. Future investigations into the role of RCD in donor TRMs during transplant rejection will deepen our understanding of macrophage heterogeneity and advance the clinical translation of precision immunomodulatory strategies targeting these cells.

In IRI, macrophages undergoing regulated cell death (such as pyroptosis or necroptosis) release DAMPs—including HMGB1, ATP, and S100 proteins—that can directly engage endothelial pattern-recognition receptors (for example, TLR4 and the NLRP3 inflammasome). This triggers upregulation of endothelial adhesion molecules (VCAM-1, ICAM-1, and E-selectin), promoting neutrophil and monocyte adhesion and transmigration, exacerbating microvascular barrier disruption and local inflammatory cascades. Moreover, the specific mode of macrophage death may further shape endothelial activation and immune–endothelial interactions by altering the composition and kinetics of DAMP release—for instance, IL-1β/IL-18 secretion during pyroptosis or MLKL-mediated membrane permeabilization in necroptosis. However, the spatiotemporal mechanisms by which distinct macrophage death subtypes regulate endothelial-specific signaling pathways in IRI remain unclear and warrant further investigation.

Macrophages dynamically modulate their antigen-presenting capacity and reshape the local immune microenvironment through RCD pathways, thereby influencing the intensity and polarization of T cell activation and adaptive immune responses. For instance, pyroptosis-associated inflammasome activation may enhance inflammatory cytokine release and dendritic cell cross-priming, while apoptosis or autophagy could differentially regulate immune tolerance versus activation states through mechanisms involving MHC-II expression or distinct patterns of DAMPs release. However, significant gaps persist in our mechanistic understanding of how different RCD subtypes exert spatiotemporally specific, microenvironment-dependent immunomodulatory effects - particularly regarding the tissue-specific regulatory roles of ferroptosis and necroptosis in immune homeostasis. Future investigations should systematically characterize the interplay between macrophage RCD modalities and adaptive immunity networks to identify novel therapeutic targets for transplantation-related immunomodulation.

In conclusion, apoptosis, pyroptosis, ferroptosis and necroptosis in macrophages exacerbate graft rejection while autophagy in macrophages protects against transplant rejection by reducing inflammation. Continued investigation is essential to clarify the precise mechanisms, signaling pathways of macrophage regulated cell death and develop effective therapeutic interventions for transplant rejection

This figure illustrates the mechanisms of various types of regulated cell death in macrophages—apoptosis, necroptosis, pyroptosis, ferroptosis, and autophagy—and their effects on organ transplantation.

Apoptosis is triggered through intrinsic (cellular stress, DNA damage) and extrinsic (extracellular signals) pathways. It involves caspases that lead to DNA fragmentation and cell membrane blebbing. Apoptosis contributes to transplant rejection by releasing damage-associated molecular patterns (DAMPs) and inflammatory cytokines. Necroptosis is initiated by TNFR binding, activating RIPK1 and RIPK3, which phosphorylate MLKL to form membrane pores, causing cell rupture. This generates significant ROS and pro-inflammatory factors, exacerbating transplant rejection. Pyroptosis is an inflammatory cell death triggered by inflammasomes that activate caspases to cleave gasdermin D (GSDMD), forming membrane pores. This leads to cell rupture and release of IL-1β and IL-18, amplifying inflammatory responses. Ferroptosis involves iron-dependent accumulation of lipid peroxides and ROS, due to impaired glutathione (GSH)-dependent repair. It promotes graft damage and rejection through oxidative stress and inflammation. Autophagy maintains cellular homeostasis by degrading damaged organelles and proteins via lysosomal activity. It involves the formation of autophagosomes that fuse with lysosomes. Autophagy protects against transplant rejection by reducing inflammation and promoting graft survival.

Overall, apoptosis, necroptosis, pyroptosis, and ferroptosis promote transplant rejection, leading to graft dysfunction. In contrast, autophagy mitigates rejection by maintaining cellular homeostasis and protecting the graft. Understanding these pathways offers insights into therapeutic strategies to improve transplant outcomes.
